# Optimized Auxin and Cytokinin Interactions Enable Direct Somatic Embryogenesis in the Peach Rootstock ‘Guardian^®^’ from Immature Cotyledons

**DOI:** 10.3390/ijms26178698

**Published:** 2025-09-06

**Authors:** Sonika Kumar, Rabia El-Hawaz, Zhigang Li, John Lawson, Stephen Parris, Foster Kangben, Lauren Carneal, Jeff Hopkins, Jacqueline Naylor-Adelberg, Jeffrey Adelberg, Gregory Reighard, Ksenija Gasic, Chalmers Carr, Christopher A. Saski

**Affiliations:** 1 Department of Plant and Environmental Sciences, Clemson University, Clemson, SC 29634, USA; rabiae@clemson.edu (R.E.-H.); zhiganl@clemson.edu (Z.L.); johndlawson320@gmail.com (J.L.); sparri2@g.clemson.edu (S.P.); fkangbe@g.clemson.edu (F.K.); lecarneal@gmail.com (L.C.); hopkin4@clemson.edu (J.H.); jnaylor@clemson.edu (J.N.-A.); jadlbrg@clemson.edu (J.A.); grghrd@clemson.edu (G.R.); kgasic@clemson.edu (K.G.); 2 Titan Farms, Ridge Spring, SC 29129, USA; chalmers@titanfarms.com

**Keywords:** *Prunus persica*, somatic embryogenesis, zygotic embryos, immature cotyledons

## Abstract

Fruit tree rootstock breeding is prolonged by extended juvenile phases, high heterozygosity, limited germplasm diversity, and hybrid incompatibilities, often requiring four decades to release new cultivars. Direct somatic embryogenesis (DSE) in established peach rootstocks presents a promising avenue for rapid genetic transformation and breeding. However, peach is highly recalcitrant to in vitro regeneration, posing major challenges for organogenesis and somatic embryogenesis (SE). This study evaluated the effects of 2,4-dichlorophenoxyacetic acid (2,4-D) and Kinetin (KIN) on SE %, SE productivity, and callus % rate in the widely used Guardian^®^ peach rootstock. A 5 × 3 full factorial completely randomized design was used to test 15 different combinations of 2,4-D and KIN on immature cotyledons, classified as upper or lower based on their position on the preculture medium. Media formulation containing a higher concentration (3.2 µM) of 2,4-D and KIN induced SE in ~50% of lower and ~85% of upper cotyledons. Optimal SE productivity occurred with higher KIN (3.2 µM) and reduced 2,4-D (2.6 µM). Callus formation peaked with 1.8 µM 2,4-D and 3.2 µM KIN. This highly reproducible research establishes a robust whole plant regeneration system via DSE in Guardian^®^ peach rootstock using immature cotyledons, providing a foundation for expedited trait manipulation through biotechnological approaches.

## 1. Introduction

Peach (*Prunus persica* (L.) Batsch) is an economically significant stone fruit species belonging to the family *Rosaceae* [1]. It serves as a genomic model for the *Rosaceae* family and was the first species within the genus *Prunus* to have its genome sequenced [2]. Peach is a diploid (2*n* = 16), with eight chromosome pairs [3], and possesses a relatively small genome size ranging from approximately 265–295 million base pairs [4]. The sequencing of the peach genome has provided valuable insights into the genetic architecture and genomic organization of the species, facilitating advanced research and breeding efforts aimed to improve the crop.

Peach is known for its relatively short juvenile period of 2–3 years in contrast to other fruit tree crops, which typically have a long juvenile phase of 6–10 years. Despite this advantage, peach breeding remains complex and challenging due to multiple factors including high heterozygosity in seed-derived offspring, limited genetic diversity, and incompatibility encountered during interspecific hybridization [5]. These challenges complicate the development of genetically stable varieties possessing desired traits through conventional breeding approaches.

Genetic transformation is a powerful biotechnological tool that has the potential to facilitate the rapid improvement of commercial cultivars by enabling the precise introduction or modification of single or multiple traits such as branch angle architecture in cotton and peach [6,7], regulation of flowering time in peach [8] and fruit related traits in tomato [9]. Advances in next-generation “omics” platforms such as genomics [10,11] transcriptomics [12,13] proteomics [14,15] and metabolomics [15,16] of peach have provided new information on candidate genes that can be targeted for functional validation through genetic transformation. Transgenic lines of fruit crops can be efficiently propagated through clonal or vegetative means, enabling the rapid production of genetically uniform plants. This capacity for large-scale multiplication enhances the utility of genetic transformation as a strategic approach for the improvement of fruit crops such as peach and for accelerating the development of novel cultivars [17].

A major obstacle to successful genetic transformation in peach is the difficulty of regenerating transformed plants. Peach, along with other *Prunus* species, exhibits high recalcitrance to in vitro regeneration via both organogenesis—the development of new organs from existing tissues and somatic embryogenesis (SE)—the formation of embryos from somatic cells [17,18,19,20]. This recalcitrance is largely attributed to the low frequency of organogenic and embryogenic responses, prolonged culture periods, high variability among regenerated plants, and low post-transplantation survival rates.

Recent advancements in SE techniques have the potential to broaden the application of biotechnology for the development of genetically modified peach plants. SE can be categorized into two pathways: direct somatic embryogenesis (DSE) and indirect somatic embryogenesis (ISE). In DSE, bipolar embryonic structures form directly on the surface of the explant without an intervening callus phase, as demonstrated in Japanese apricot [21] and olives [22,23]. In contrast, ISE involves an initial callus induction step, followed by the differentiation of embryos from the callus tissue, a process reported in species such as cotton [24], oak [25], *Arabidopsis* [26], radiata pine [27], and eucalyptus [28].

Several studies have proposed the use of seed-derived explants, including immature and mature zygotic embryos, to induce SE in peach. However, the efficiency of SE induction remains highly dependent on multiple factors, such as genotype, seed size, and the developmental stage of seed maturation [29,30,31]. Juvenile explants, particularly immature embryos, are often favored due to their enhanced proliferative capacity and greater competence for somatic cell division compared to mature tissues. To date, reports of SE in peach using immature tissues remain limited. The first documented instance of SE in peach was reported by Hammerschlag and his group in 1985 [32], who achieved embryogenesis from friable callus derived from immature embryos. Subsequently, in 1991 Bhansali and his team [33] successfully induced SE using cotyledonary slices, further demonstrating the potential of immature tissue explants for regeneration in peach. SE has also been initiated from immature zygotic embryos of several peach genotypes, including ‘Belle of Georgia’, ‘Bailey’, ‘Tennessee Natural’, ‘Nemaguard’ and ‘Encore’ [31]. Somatic embryos were also induced from immature cotyledons (50 to 70 days post-bloom) in ‘O’Henry’, ‘Elegant Lady’, ‘Rich Lady’, and ‘Venus’ [30].

To date, there are no confirmed reports of successful SE from mature peach tissues. The induction of SE from both mature and immature tissues is influenced by a range of factors, including the type of explant, culture conditions, hormonal treatments, and genotype. Recently, Ricci and his team [30] evaluated the SE potential of mature tissues, including young leaves derived from in vitro meristematic bulks of the commercial peach rootstock ‘Hansen 536’ (*P. persica* × *P. dulcis*). However, these attempts did not result in the formation of somatic embryos. Similarly, petals and anthers from unopened flowers of the peach rootstock ‘GF677’ (*P. persica* × *P. dulcis*) were tested for SE induction but also failed to exhibit embryogenic potential [30,34].

Phytohormones and their interactions play a critical role in regulating SE and the regenerative capacity of embryogenic tissues. The balance and cross talk between auxins and cytokinins, through both synergistic and antagonistic interactions, are central to the induction and progression of SE [35,36]. Among auxins, 2,4-dichlorophenoxyacetic acid (2,4-D) is particularly important for initiating embryogenic responses, while among cytokinins, KIN and 6-benzylaminopurine (BAP) are commonly employed to support SE induction in a variety of crop species, including peach and nectarine [37], Japanese apricot [21], grapevine [38], and rice [39]. 2,4-D plays a critical role in the induction of SE and the early stages of embryo development; however, extended exposure to 2,4-D can negatively affect embryo maturation, regeneration efficiency, and overall productivity [40]. Cytokinins contribute significantly to embryo maturation, germination, and subsequent plant regeneration. The optimal auxin-to-cytokinin ratio necessary for successful SE is highly dependent on species, explant type, and genotype, and must be empirically determined for each system.

The development of peach cultivars for use as rootstocks through conventional breeding is a lengthy and complex process, often requiring up to 40 years depending on breeding objectives, multi-environment field trials, and the suite of targeted traits. The Guardian^®^ peach rootstock has become widely adopted in the southeastern United States due to its proven resistance to the peach tree short life (PTSL) disease complex, vigorous growth, and favorable horticultural characteristics. Nicknamed the “guardian angel” for its protective role against PTSL, Guardian^®^ was released in 1993 [41,42,43]. Despite its success in mitigating PTSL, Guardian^®^ remains highly susceptible to *Armillaria* root rot (ARR), a devastating disease affecting economically important stone fruit and nut crops across the United States. This susceptibility, coupled with its otherwise strong agronomic profile, makes Guardian^®^ an ideal candidate for targeted improvement through genetic engineering and genome editing technologies.

Notably, in the more than three decades since its release, there have been no published reports documenting successful DSE or ISE in Guardian^®^ from either mature or immature tissue. Although San and his group [44] established a regeneration system using leaf explants of Guardian^®^ in 2015, their methodology relied on organogenesis, not SE, and lacked reproducibility. Similarly, prior experiments conducted in the Saski lab at Clemson University tested various explants including leaves, stems, shoot apical meristems, and mature seeds for SE in *Guardian*^®^, but these attempts were unsuccessful, likely due to the lack of juvenility in the explants. These results suggested that immature cotyledons, because of their juvenile nature, could serve as a more suitable explant source for SE. However, the use of immature cotyledons also presents challenges, including their limited availability throughout the year, the critical need to select the correct developmental stage of the zygotic embryos, and generally low SE efficiency.

To address these challenges, we developed an efficient and reproducible protocol for DSE from immature cotyledons of Guardian^®^ peach and achieved continuous maintenance of somatic embryos throughout the year. This protocol utilizes varying concentrations of the plant growth regulators (PGRs) 2,4-D and KIN to optimize embryogenic response. The regeneration of whole plants via DSE and their preliminary evaluation are presented, establishing a foundational system for future genetic transformation and trait improvement efforts in this commercially valuable rootstock.

## 2. Results

A pilot experiment was conducted in 2021 to investigate the process of SE in Guardian^®^ peach rootstock using somatic embryogenesis induction media (SEIM) supplemented with a 1:1 ratio of 2,4-D and KIN with 2.0 µM concentration. Somatic embryos were developed after 40 days of subculturing and were regenerated into whole plants. Based on these results, a 5 × 3 full factorial experiment with completely randomized design was conducted to optimize media composition using varying concentrations of 2,4-D and KIN for DSE in peach (Table 1). To assess the impact of 2,4-D and KIN on SE induction, several response parameters were evaluated, including SE %, SE productivity, and callus %.

### 2.1. Effect of PGRs on Somatic Embryogenesis Rate (SE %)

**Figure 1:**
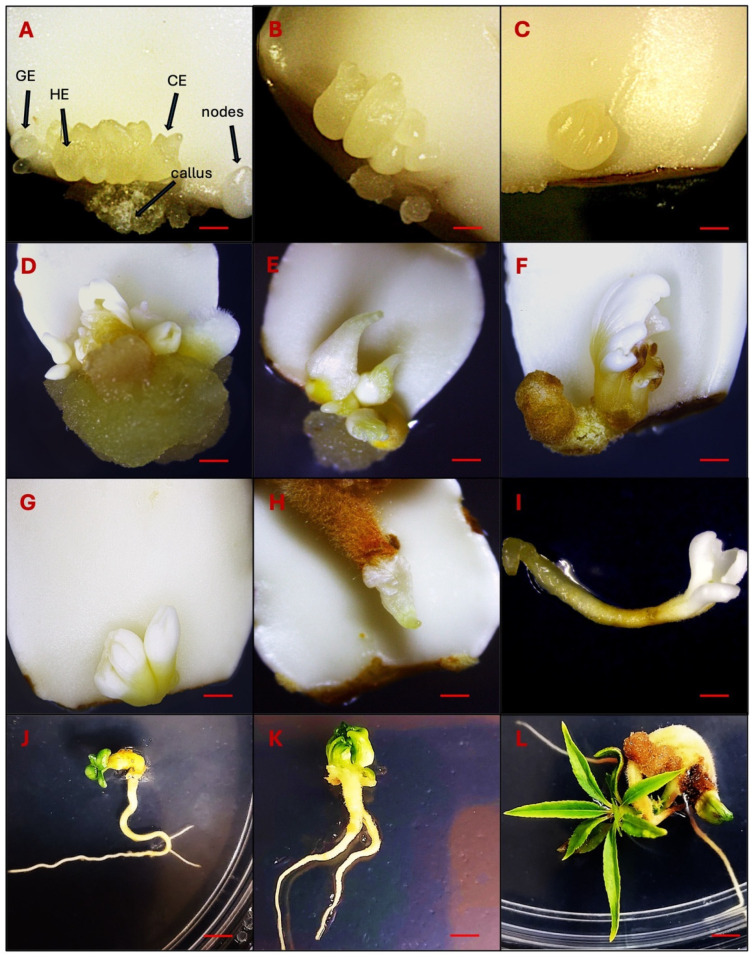
Observations of DSE at different developmental stages. (**A**–**C**): Direct somatic embryo development on the adaxial surface on the proximal end of immature cotyledons 30–40 days after induction (Globular embryo-GE, Heart embryo-HE and Cotyledonary embryo-CE). (**D**–**G**): Somatic embryo differentiation 80–120 days after induction. (**H**–**L**): Germinating somatic embryos 150 days after induction. Bars: (**A**–**C**) = 1000 µm, (**D**–**I**) = 500 µm, (**J**–**L**) = 300 µm.

DSE was observed 25–30 days after culturing immature cotyledons on SEIM (Figure 1A–C). Somatic embryos, characterized by their white, shiny, and globular appearance, developed on the adaxial surface near the proximal end of the cotyledon. These somatic embryos could be readily differentiated from the calli, as calli were yellow or brown in color and softly textured (Figure 1A–C). The SE % was influenced by various factors, with the three most significant being 2,4-D × KIN interaction, KIN, and 2,4-D (Table 2A). Other significant factors were cotyledon location, KIN × cotyledon location interaction, 2,4-D × 2,4-D interaction and 2,4-D × cotyledon location interaction. A positive interaction was observed between 2,4-D and KIN, contributing to the successful induction of SE and observed that the combined effect of 2,4-D and KIN influences SE % differently than their individual effects.

**Figure 2:**
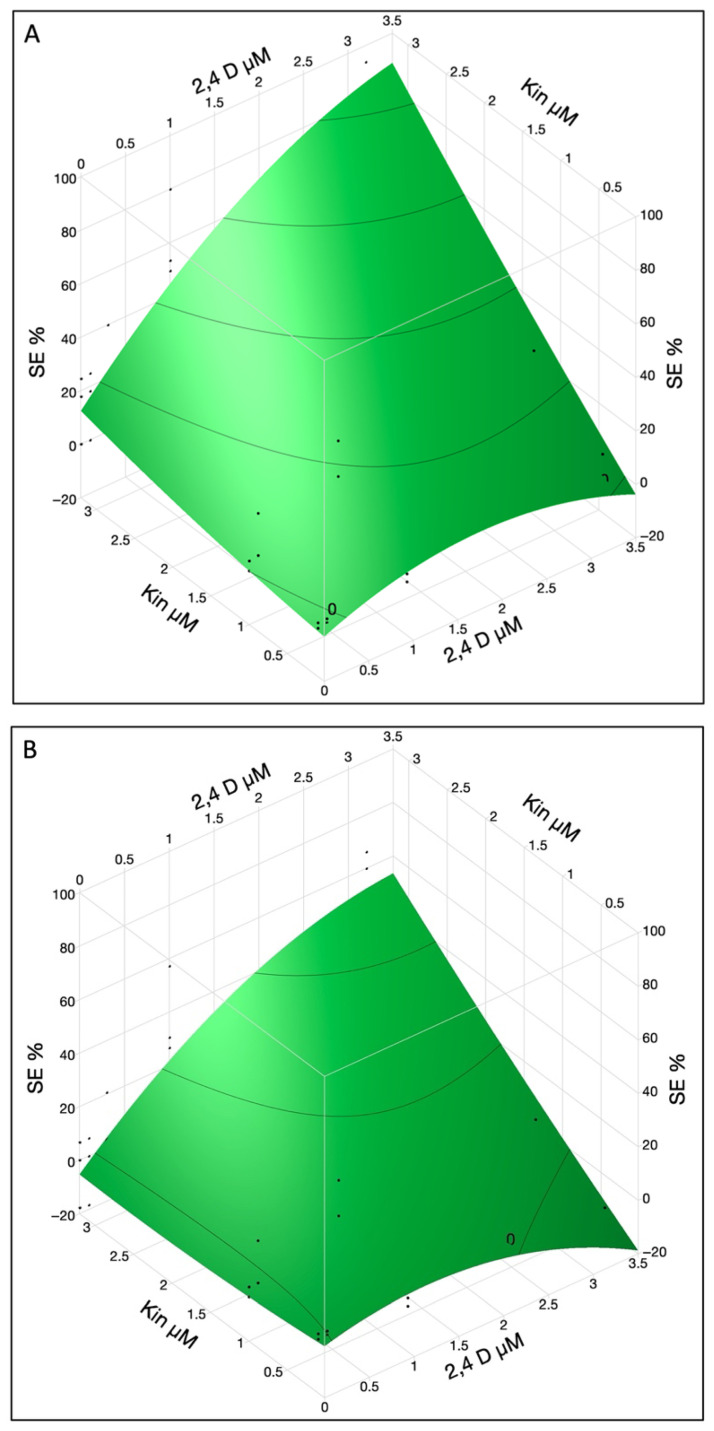
The SE % in immature cotyledons was affected by interaction of 2,4-D and KIN in upper (**A**) and lower cotyledons (**B**). The contour surface was presented with the surface plus residual points.

Furthermore, the cotyledon location also exhibited interactions with both hormones, resulting in an enhanced rate of SE. High concentration (3.2 µM) of 2,4-D and KIN induced a higher percentage of SE, i.e., ~50% and ~85% for both the lower and upper cotyledons, respectively (Figure 2A,B). To maximize the SE %, a higher concentration of KIN (>3.2 µM) can be used while 2,4-D at 3.2 µM concentration seems to be optimal for SE induction in both lower and upper cotyledons.

### 2.2. Effects of PGRs on SE Productivity

SE productivity stands as a crucial parameter in the proliferation of peach somatic embryos. Among the factors considered, KIN had the most substantial impact on the number of somatic embryos produced from productive explants, followed by 2,4-D, 2,4-D × KIN interaction, 2,4-D × 2,4-D interaction and cotyledon location (Table 2B).

**Figure 3:**
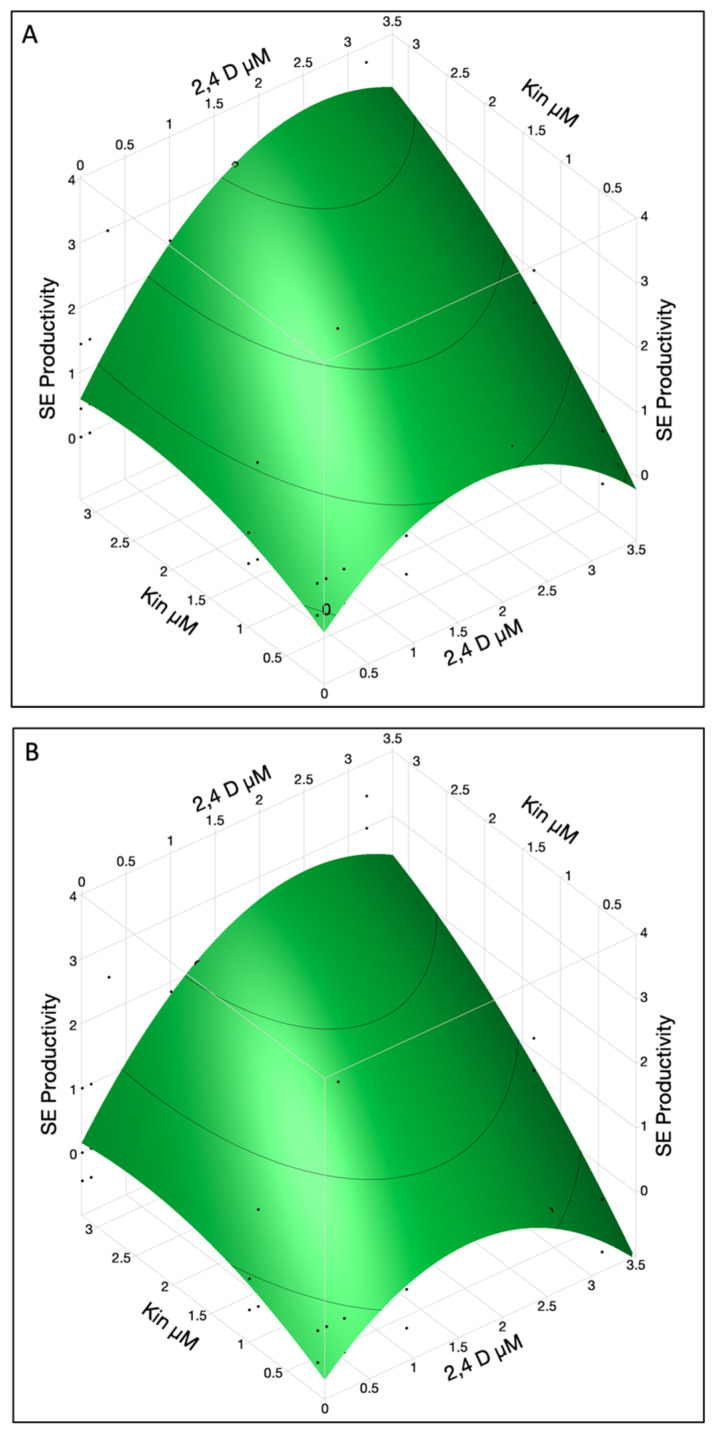
The SE productivity in immature cotyledons was affected by interaction of 2,4-D and KIN in upper (**A**) and lower cotyledons (**B**). The contour surface was presented with the surface plus residual points.

A combination of 2,4-D and KIN showed a good response to SE productivity in upper and lower cotyledons (Figure 3A,B). In lower cotyledons maximum SE productivity was observed when 2,4-D conc was ~2.6 and KIN was used at a rate of 3.2 µM while in the upper cotyledon SE productivity value was ~3.4. It was observed that 2,4-D reached its maximum level at 2.6 and KIN still showed the linear terms so more KIN can be used for increased productivity.

High concentration of 2,4-D (3.2 µM) was not desirable for SE productivity as it resulted in an excessive callus growth instead of embryo differentiation. To obtain the maximum SE productivity, a higher KIN concentration (>3.2 µM) can be used while 2,4-D at 2.6 µM concentration was optimum for both lower and upper cotyledons. In summary, the productivity of SE is influenced by factors such as KIN, 2,4-D, and cotyledon location. The interaction between 2,4-D and KIN seems to amplify their individual effects, enhancing SE productivity.

### 2.3. Effect of PGRs on Callogenesis Rate (Callus %)

A high level of callogenesis was observed in both the upper and lower cotyledons with final data recorded after 80 days of subculturing. Following a 5-week inoculation period, slight callus formation was observed primarily at the proximal cut ends of the explants. However, in some cases, calli also emerged at both the proximal and distal cut ends of the explants. After 80 days of inoculation, a soft-textured, non-embryogenic callus was formed, displaying colors that varied from yellow to brown (Figure 1D,E).

**Figure 4:**
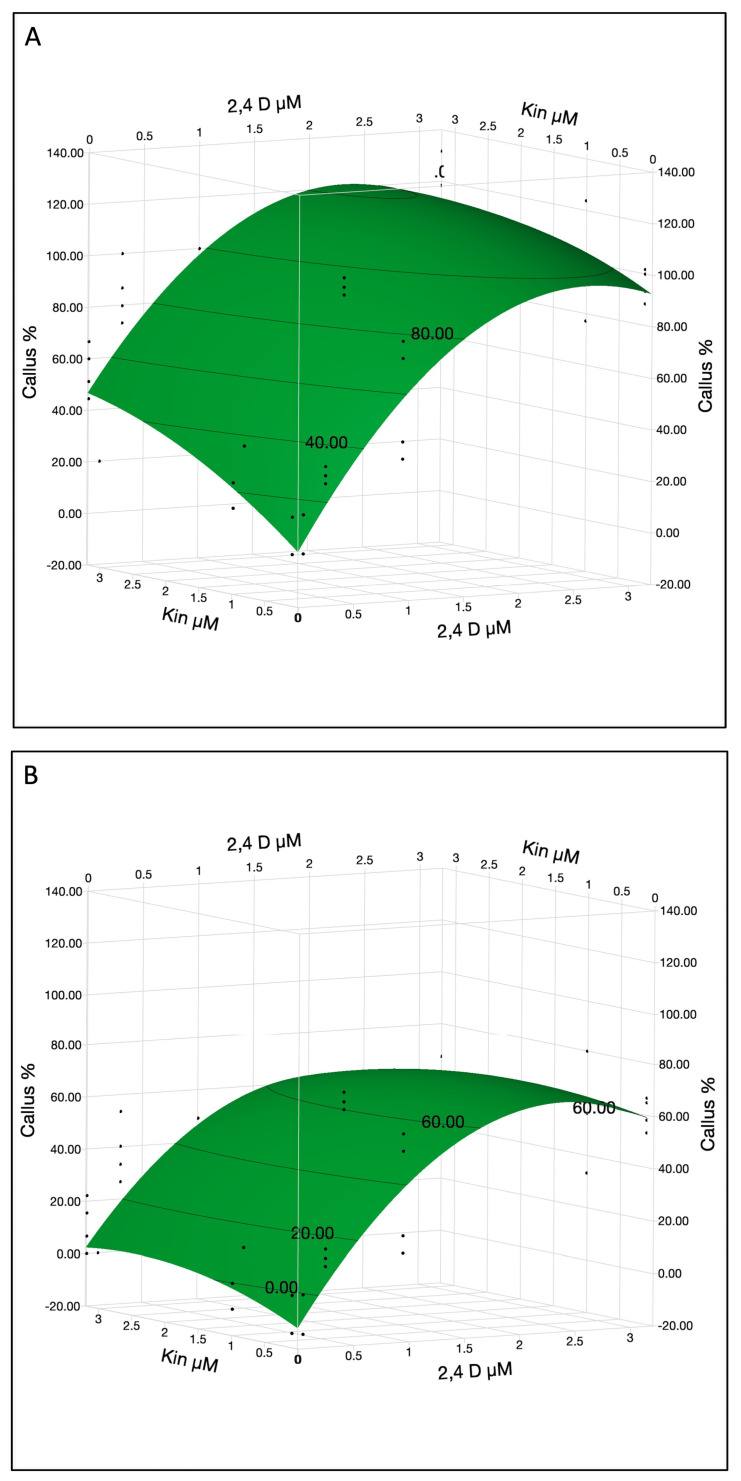
The callus % in immature cotyledons was affected by 2,4-D and KIN in upper (**A**) and lower cotyledons (**B**). The contour surface was presented with the surface plus residual points.

The most significant factors influencing the callogenesis rate were 2,4-D, cotyledon location and 2,4-D × 2,4-D interaction (Table 2C). Other significant factors were KIN, KIN × cotyledon location interaction and 2,4 D × KIN interaction. KIN at 3.2 µM was optimal for callus formation while 2,4-D curve showed quadratic effects and optimum concentration was 1.8 µM for both lower and upper cotyledons with ~60% and ~100% callus formation efficiency, respectively (Figure 4A,B). However, the plotting function illustrates predicted callus percentages up to 120% callus in upper cotyledons, which represent model generated predictions rather than empirical measurements. The surface plot displays predictions derived from the fitted model, not actual observed data. Callus percentage values exceeding 100%, as seen for upper cotyledons, indicate peak model responses and should not be interpreted as literal percentages.

Callus formation was optimized at ~1.8 µM of 2,4-D and 3.2 µM KIN. Excessive concentration may not significantly improve callus formation. These results confirm that 2,4-D and KIN play a crucial role in the induction of calli from immature cotyledon; however, these calli were non-embryogenic and did not produce embryos even after an 80-day period of inoculation.

### 2.4. Somatic Embryos Maturation, Germination and Acclimatization

The process of somatic embryos maturation has significant importance in SE. When somatic embryos differentiated into the cotyledonary stage, they were transferred to differentiation media (DM) for maturation, i.e., the development of shoots and roots (Figure 1D–H). In the present experiment, we achieved success in transitioning somatic embryos into complete plantlets. Though, the germination and conversion rate remained notably low, reaching only 10%. The somatic embryos germinated after 120–140 days of induction (Figure 1I–L).

**Figure 5:**
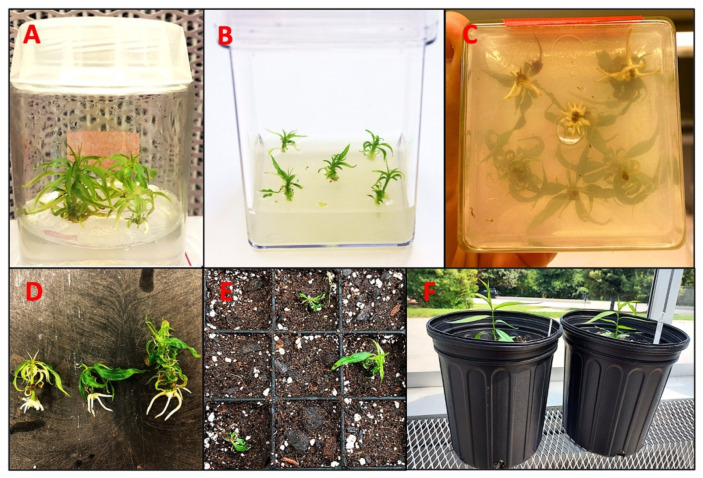
Rooting of regenerated Guardian^®^ shoots. (**A**) Shoot propagation on QL media. (**B**) Shoots transferred on rooting media. (**C**) Rooted shoots in magenta box. (**D**) Rooted shoots before transferring to soil. (**E**) Seedling transferred to soil, kept in a mist chamber. (**F**) Acclimatized seedlings in green house.

Nevertheless, certain somatic embryos failed to germinate or develop roots. To address this issue, an alternative rooting protocol was utilized, ultimately facilitating the conversion of somatic embryos into fully developed plants (Figure 5A–F). In this protocol, non-germinated somatic embryos were first propagated on QL media, which is used for shoot propagation in Guardian^®^. The propagated shoots were subsequently transferred to rooting media to initiate root development. Finally, rooted plantlets were transplanted into soil and acclimatized.

**Figure 6:**
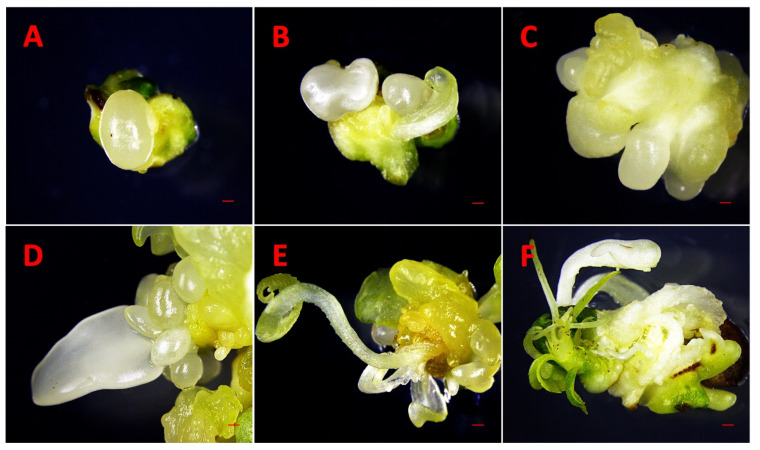
Initiation of secondary somatic embryos. (**A**) Globular shape. (**B**) Heart shape. (**C**) Torpedo shape. (**D**) Cotyledonary shape. (**E**,**F**) Mature cotyledonary somatic embryos and leaves formation. Bars. (**A**–**F**) = 500 µm.

For long-term maintenance, somatic embryos were cultured on DM but kept in the dark. We saw the rapid development of secondary somatic embryos directly from the old somatic embryos without the formation of calli (Figure 6A–F). Secondary somatic embryos were bigger in size and developed in clusters. These secondary somatic embryos were maintained and proliferated for a long duration after 30 days of subculturing to fresh media under the dark.

## 3. Discussion

Previous studies have consistently reported that peach is highly recalcitrant to in vitro regeneration through both organogenesis and SE, posing a significant barrier to biotechnological applications in this species [17]. Despite this, several studies have demonstrated the feasibility of regenerating peach plants from a variety of explant sources. Successful regeneration has been reported from immature endosperm [45], zygotic embryo-derived callus cultures [32,37,46], immature cotyledons [37,47] mature cotyledons [5,48] leaf explants [49], and hypocotyl slices [5]. These studies collectively suggest that while a range of explant types can be employed for in vitro regeneration in peach, the efficiency of regeneration is highly variable and strongly influenced by both the explant type and the specific culture conditions employed.

Despite these advances, several technical challenges continue to limit the routine application of in vitro regeneration in peach. Among the most persistent issues are the low frequency of organogenic and somatic embryogenic responses, high variability among regenerants, and poor survival rates following plantlet transplantation. These limitations underscore the need for continued optimization of explant selection, culture media composition, and regeneration protocols to improve reliability and efficiency in peach tissue culture systems. In the present study, we established a highly reproducible plant regeneration protocol for the peach rootstock cultivar Guardian^®^ using a DSE approach from immature cotyledons. To our knowledge, this represents the first report of an in vitro regeneration system via SE for Guardian^®^, a widely adopted rootstock in the southeastern United States using immature cotyledons.

Initial pilot experiments conducted in 2021 indicated that both the developmental stage of the seed and the size of the immature cotyledons are critical factors influencing the induction of SE in this genotype. Immature zygotic embryos are known to exhibit greater responsiveness to SE induction compared to mature embryos in various crop species such as *Arabidopsis* [26], Japanese apricot [21], and tea oil plant [50]. This increased embryogenic potential is typically attributed to their higher mitotic activity and developmental plasticity. Genotypic variation is also a well-documented factor influencing SE response, with some peach genotypes showing a markedly higher capacity for organized embryogenic structure formation than others [18,30,31,37]. While the current study focused exclusively on Guardian^®^, the protocol developed here provides a valuable framework that may be adapted and tested across additional *Prunus* genotypes for broader applicability in peach biotechnology and breeding programs.

PGRs are known to play a crucial role in regulating SE potential across diverse crop species. Successful regeneration through SE requires precise optimization of both the type and concentration of PGRs. The balance between auxin and cytokinin is critical in directing the developmental fate of cultured cells. Auxins such as 2,4-D promote callus induction and induce embryogenic potential, but excessive levels can maintain tissues in an undifferentiated state. Reducing auxin after callus induction often favors embryo development and supports healthy plant regeneration. Cytokinins like KIN stimulate organized cell division, but excessive levels may redirect development toward organogenesis rather than embryogenesis. In general, high auxin concentrations favor callus formation, moderate auxin with balanced cytokinin supports somatic embryogenesis, and disproportionate cytokinin promotes shoot formation. Thus, both concentration and ratio are crucial for the successful regeneration of whole plants. Consistent with prior reports in peach [31,37], auxins and cytokinins were utilized either individually or in combination to induce SE in this study. Globular stage somatic embryos began to appear between 25–30 days after culture initiation. Interestingly, SE induction was significantly more efficient in the upper cotyledons compared to the lower cotyledons, despite the latter having greater physical contact with the medium.

An interesting synergistic interaction between 2,4-D and KIN was observed, with the combined application of both PGRs resulting in higher SE induction rates than either hormone alone. Additionally, the location of the cotyledon on the explant surface influenced responsiveness, with hormone-explant interactions contributing to enhanced SE rates. These findings are in agreement with previous reports in other species, such as orchid [51] and tea oil plant [50], where spatial orientation and hormonal cross-talk significantly influenced embryogenic outcomes. Taken together, these results highlight the importance of both explant positioning and the specific ratio of 2,4-D to KIN in optimizing SE induction and whole plant regeneration in the Guardian^®^ peach rootstock.

Our findings are consistent with previously published studies on SE in peach and other plant species. For example, Srinivasan and Scorza [31] investigated the effects of various auxins, including 2,4-D, picloram, dicamba, and centrophenoxine, in combination with KIN and BAP, on SE induction in peach. Among these treatments, 2,4-D at a concentration of 25 μM was the most effective in initiating somatic embryos. However, despite successful induction, they were unable to regenerate complete plants. This suggests that excessively high concentrations of 2,4-D may impair the regenerative capacity of somatic embryos, possibly by disrupting hormonal balance or embryonic development.

Our results corroborate the broader literature indicating that 2,4-D is one of the most effective auxins for SE induction across multiple plant species, including cotton [24], tea oil plant [50], grapevine [38], Japanese apricot [21], and peach [37]. In the current study, we observed that the combination of 2,4-D and KIN induced somatic embryos in approximately 50% of lower cotyledons and up to 80% of upper cotyledons, demonstrating a high frequency of embryogenic response under optimized conditions.

These findings are also in agreement with reports from other systems where auxin-cytokinin combinations, particularly 2,4-D with either KIN or BA, have been successful in promoting SE. For example, somatic embryos were efficiently induced in Japanese apricot using 2,4-D and KIN [21], and similar hormonal combinations were effective in model legumes [52], grapevine [34], Burma reed [53], and the tea oil plant [50]. Collectively, these results reinforce the critical role of auxin-cytokinin interactions in SE and highlight the applicability of our protocol for peach rootstock Guardian^®^ within the broader context of woody perennial crop improvement.

The combined application of 2,4-D and KIN appears to exert a synergistic effect on the induction of SE and subsequent regeneration in peach, particularly in the Guardian^®^ rootstock. This synergism suggests that the interaction between these two PGRs enhances embryogenic competence more effectively than either regulator applied alone. The improved response likely reflects the complementary roles of auxins and cytokinins in promoting cell division, dedifferentiation, and the developmental transition to embryogenesis. Understanding the interplay between 2,4-D and KIN, as well as their optimal concentrations and ratios, is critical for refining SE protocols in peach. Such insights are essential not only for improving the efficiency of in vitro regeneration systems but also for enabling downstream applications in genetic transformation and genome editing. As SE serves as a foundational platform for biotechnological interventions, optimizing hormonal interactions represents a key step toward the propagation and genetic improvement of recalcitrant woody species like peach.

**Figure 7:**
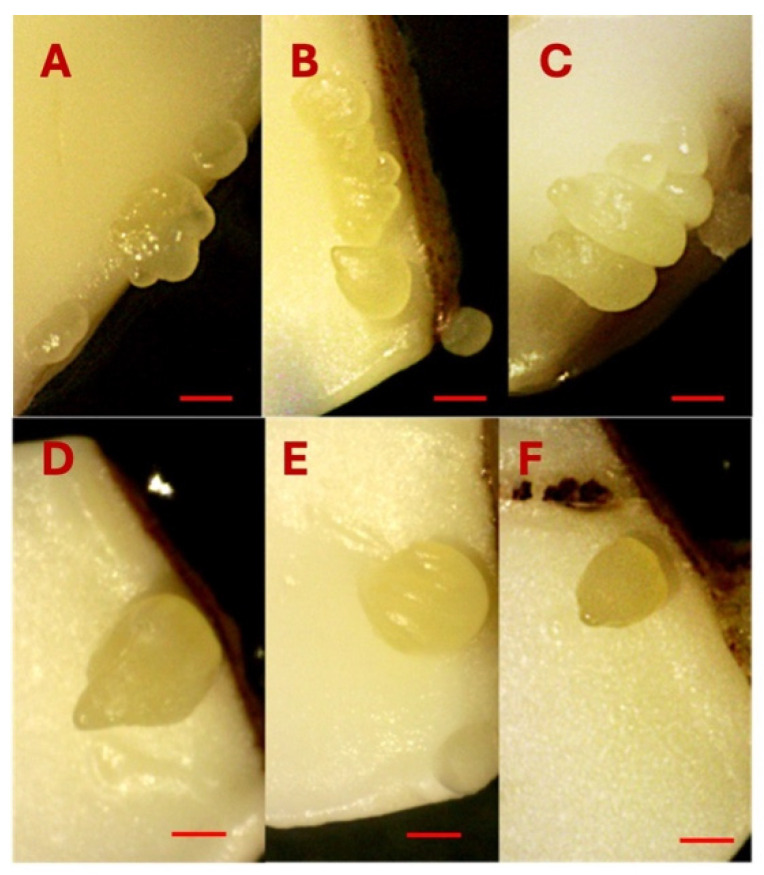
Multiple somatic embryo induction on the cotyledons (**A**–**C**). Single somatic embryo induction on the cotyledons (**D**–**F**). Bars: (**A**–**F**) = 1000 µm.

Somatic embryos were induced directly from the cut surface, specifically the proximal end of the explants, suggesting that cells located near the proximal region possess a higher embryogenic potential compared to those at the distal end. In the majority of explants, somatic embryos developed in clusters, with 2 to 7 embryos observed per explant (Figure 7A–C). However, in some media combinations with low 2,4-D concentration (0.01–0.10 µM) and high KIN (3.2 µM), a single somatic embryo was also formed (Figure 7D–F). The combination of 3.2 µM 2,4-D and KIN induced a good response to SE productivity in upper and lower cotyledons, leading to the formation of multiple embryos (>3) per cotyledon.

These somatic embryos progressed through the complete developmental sequence, including globular, heart, torpedo, and cotyledonary stages (Figure 1A–F), indicating a well-differentiated embryogenic pathway. The somatic embryos were loosely attached to the cotyledon surface, allowing for easy detachment and subsequent transfer to differentiation media for shoot and root development. Importantly, the frequency of somatic embryo induction was significantly higher in the upper cotyledons than in the lower cotyledons. This difference may be attributed to the absence of direct contact between the upper cotyledon and the culture medium, which likely creates a localized stress environment conducive to SE. These observations align with the widely accepted hypothesis that somatic embryos formation can be triggered as a stress-induced response, mediated by PGRs or by biotic, abiotic, and environmental stress factors [54].

**Figure 8:**
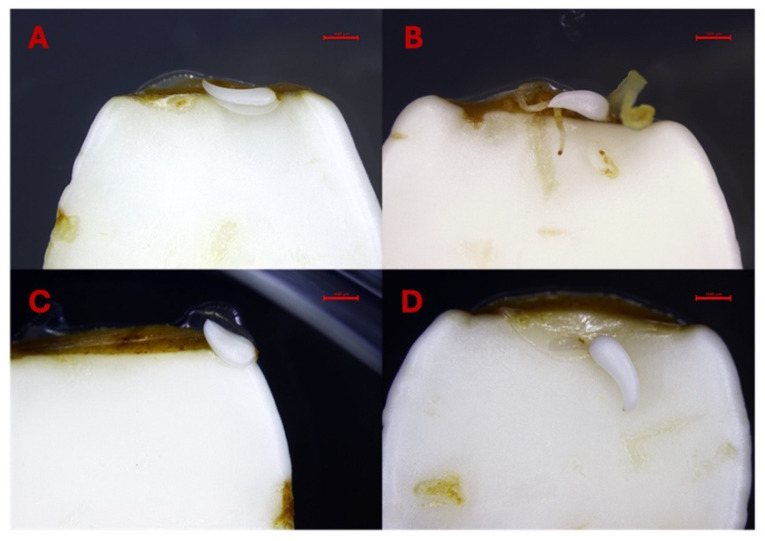
(**A**–**D**). Formation of bracts on the lower cotyledons. Bars: (**A**–**D**) = 1000 µm.

**Figure 9:**
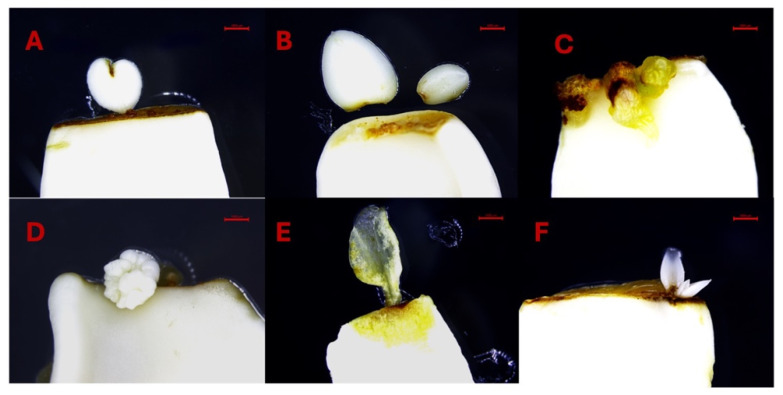
(**A**–**F**). Unusual tissue and abnormal somatic embryo formation on lower cotyledons. Bars: (**A**–**F**) = 1000 µm.

The results underscore the importance of explant orientation and microenvironmental conditions in optimizing SE, particularly in recalcitrant species such as peach. The lower cotyledons exhibited more growth in size on the preculture media in contrast to the upper cotyledons. In the lower cotyledons, we also observed the formation of leaf bracts (Figure 8A–D), likely due to direct contact with the culture medium. This contact provided optimal, non-stressful conditions with sufficient nutrient availability, promoting leaf bract development rather than somatic embryos. Additionally, alongside typical somatic embryos, some treatments induced the formation of atypical tissues and abnormal somatic embryos which appeared to be related to individual explant variation rather than hormone concentration (Figure 9A–F).

In this study, a high frequency of callogenesis was observed, and this response was quantitatively assessed as callus %. Despite the widespread induction of callus, all calli were non-embryogenic and did not give rise to somatic embryos. Non-embryogenic callus typically originated from the proximal end of the explant approximately 40 days after induction, although in some cases, callus formation was also observed at both the proximal and distal ends. The extent and morphology of callus formation varied considerably among different media treatments. In some conditions, callus tissues expanded substantially over an 80-day culture period, whereas in others, only minimal callus growth was observed. Importantly, a distinct pattern was noted that differentiated embryogenic from non-embryogenic responses in explants. Embryogenic explants successfully underwent SE, with somatic embryos formation preceding callus development, whereas in non-embryogenic explants, only callus formation occurred with no evidence of subsequent somatic embryos initiation. Additionally, some explants exhibited the formation of small callus masses along the cut surfaces, which eventually turned brown and ceased to proliferate. This browning is likely indicative of cellular senescence or necrosis, commonly associated with oxidative stress or prolonged culture without differentiation. Collectively, these findings emphasize the importance of distinguishing between embryogenic and non-embryogenic callus and optimizing culture conditions to favor DSE, while minimizing non-productive callus responses.

Somatic embryos were effectively maintained and multiplied on differentiation medium supplemented with 1 μM naphthaleneacetic acid (NAA) and 5 μM BAP. This medium demonstrated high efficacy in promoting the formation of secondary somatic embryos, supporting continuous embryogenic activity. Subculturing was required at 30-day intervals, indicating the suitability of this formulation for long-term culture maintenance. In our study, secondary somatic embryos (Figure 6A–F) developed rapidly and were consistently larger than the old somatic embryos, a trend previously observed in peach and nectarine [37] and in Japanese apricot [21].

**Figure 10:**
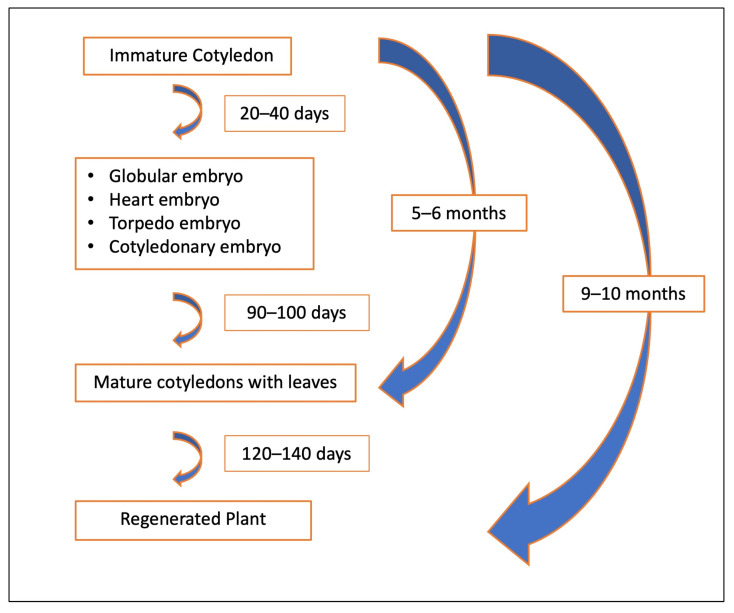
Timeline for direct somatic embryogenesis system in Peach.

Somatic embryos, whether occurring individually or in clusters and attached to the cotyledon, were carefully detached and transferred to differentiation medium to promote germination. A portion of these somatic embryos successfully germinated, forming both shoots and roots (Figure 1I,J). However, a subset of somatic embryos exhibited incomplete germination, producing shoots without roots. These shoot-only regenerants were maintained and propagated using QL medium [55] while root induction was achieved using an optimized rooting procedure for peach [56] (Figure 5A–F). The partial germination and rooting difficulties observed in this study are consistent with earlier reports indicating that the successful conversion of peach somatic embryos into complete plantlets remains a challenging process. These challenges persist despite efforts to optimize growth media and tightly control microenvironmental conditions [18,31,46]. Our findings reinforce the need for continued refinement of post-embryogenic development protocols to improve the efficiency of plantlet recovery in peach regeneration systems. Our approach using immature cotyledons to induce DSE has led to the successful regeneration of peach plants in 9–10 months (Figure 10).

This method is in line with findings by von Arnold and his group [57] and other researchers who have highlighted the advantages of DSE over ISE. DSE is faster than ISE and requires less genetic reprogramming, reducing the possibility of somaclonal variation or the regeneration of abnormal plants.

## 4. Materials and Methods

### 4.1. Plant Material and Execution of Inoculation of Explants

**Figure 11:**
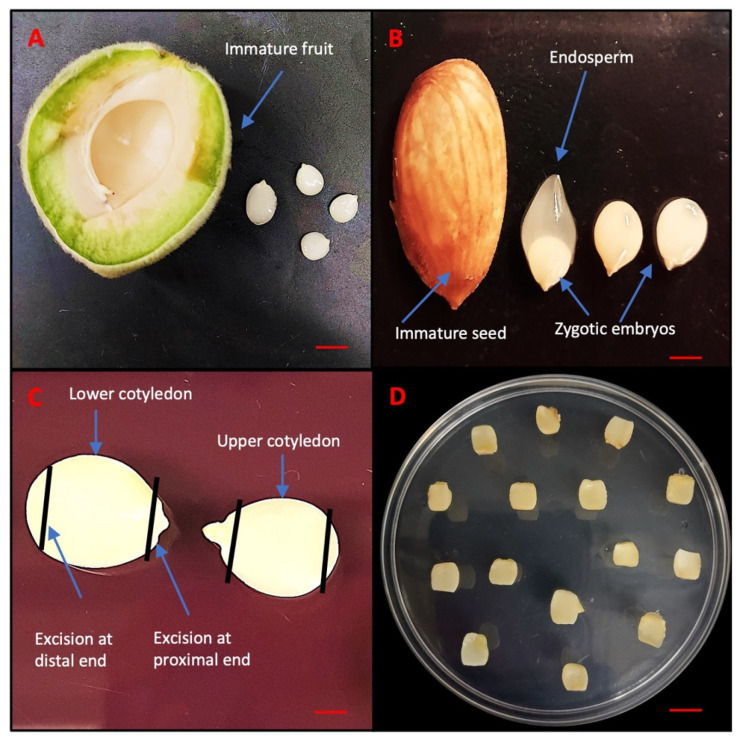
Sampling of different zygotic embryos of peach rootstock Guardian^®^. (**A**) Immature fruit. (**B**) Immature seed and zygotic embryos. (**C**) Excision of immature cotyledons. (**D**) Immature cotyledons inoculated in MS medium. Bars: (**A**,**B**) = 5 mm, (**C**) = 1 mm and (**D**) = 10 mm.

In this study, all media formulations, hormones, and other chemicals were used from PhytoTech Labs, Lenexa, KS, USA, unless otherwise specified. Immature fruits of Guardian^®^ (4–6 cm length) were collected 30 days after pollination from five different trees grown at the Musser Fruit Research Center, Clemson University, Seneca, SC, USA and stored at 4 °C (Figure 11A). For surface sterilization, the immature fruits were first treated with 70% (*v*/*v*) ethanol (PHARMCO by Greenfield Global, Toronto, ON, Canada) for 10 min outside the hood, followed by a 20 min treatment with 20% (*v*/*v*) commercial bleach (5.25% (*w*/*v*), sodium hypochlorite) containing 0.1% (*v*/*v*) Tween 20. Then, the fruits were rinsed three times with sterile distilled water. The next step was performed inside the hood and involved manually removing the endocarp using clippers and collecting 15 seeds in falcon tubes to prevent contamination. These immature seeds, which contained the zygotic embryo (3–5 mm length) enclosed inside the endosperm (Figure 11B), were subjected to another round of surface sterilization using 15% bleach for 3 min and rinsed three times with sterile distilled water. The zygotic embryos were carefully excised from the seeds under aseptic conditions. These embryos were placed horizontally in 90 mm × 15 mm disposable Petri dishes (VWR international, LLC, Radnor, PA, USA) containing 30 mL of preculture media known as Quoirin and Lepoivre (QL) [58] without hormone. To avoid any contaminated explants, a preculture period of 7 days was maintained in the dark at approximately 24 °C. On preculture media, the zygotic embryos grew up into two distinct cotyledons differing in their size. The lower cotyledon, in contact with the media, grew larger than the upper cotyledon, which remained small due to lack of direct contact with the media (Figure 11C). For the experimental setup, cotyledons were designated as upper and lower and cultured on embryo induction media.

### 4.2. SE Induction

Based on a previous study [21], Murashige and Skoog (MS) [59] media was chosen as the basal culture media for the DSE in peach immature cotyledons. The MS media used in the experiment was supplemented with 3% (*w*/*v*) sucrose, B5 vitamins and solidified with 2.6% (*w*/*v*) phytagel, and the pH was adjusted to 5.8 before autoclaving at 121 °C for 20 min. Based on the 5 × 3 full factorial completely randomized design, different concentrations of 2,4-D (0.01, 0.10, 0.30, 1.00, 3.20 μM) and KIN (0.10, 1.00, 3.20 μM) were added using a log_10_ scale, and 50 mL media was poured in 90 mm × 25 mm disposable Petri dishes (VWR international, LLC, Radnor, PA, USA). A total of 15 different combinations involving varying proportions of 2,4-D and KIN were tested for induction of SE and termed as somatic embryogenesis induction media (SEIM) listed in Table 1. However, 2,4-D concentration was also tested at very low concentration (0.01) to see the individual effect of KIN on somatic embryogenesis. To evaluate the induction of somatic embryos, each explant was excised at both the proximal and distal ends. Figure 11C. A total of 15 explants designated as upper and lower cotyledons were placed on each Petri dish and the entire set of 15 treatments were replicated (Figure 11D). The Petri dishes were then sealed with saran wrap and kept in the dark at a constant temperature of approximately 24 °C. Explants were subcultured into the same treatment media after 40 days.

### 4.3. Parameters Selection and Recording

After 80 days following initial sub-culture, data were collected on SE, including SE %, SE productivity, and callus %. These parameters were evaluated to determine the efficiency of SE across different treatment conditions. Calculations for each parameter were as follows:

SE % = (Total number of explants producing somatic embryos/Total number of explants cultured per plate) × 100

SE productivity = Total number of somatic embryos produced per plate/Total number of explants forming somatic embryos per plate

Callus % = (Total number of explants producing callus/Total number of explants cultured per plate) × 100

### 4.4. Maturation and Germination of SEs and Plant Acclimatization

After collecting data on the desired parameters, all the explants with somatic embryos were transferred from the SEIM to the DM and kept in the dark. The DM consisted of an MS medium containing 0.1 μM NAA and 5.0 μM BAP [21]. During the differentiation stage, some of the somatic embryos started to germinate and developed roots. These germinated somatic embryos were carefully separated from the explants, transferred to QL media, and maintained at the temperature of 24 ± 1 °C under a 16/8 h day/night cycle with a light intensity of 60 µmol m^−2^ s^−1^ until they developed into intact plants. The process involved nurturing the germinated embryos on the QL media until they grew into fully formed plants with well-established root systems.

However, in some cases, certain somatic embryos failed to develop roots, although their shoots were fully formed. These developed shoots were propagated on QL medium, and rooted plants were subsequently generated and acclimatized using a rooting protocol [56].

### 4.5. Morphological Observations

Morphological observations of different developmental stages of Guardian^®^ were taken from EOS Rebel T7 Cannon digital camera (Cannon, Melville, NY, USA), Dino-Lite Edge 3.0 Microscope (Dino-Lite, Torrance, CA, USA) and Leica M125 Stereoscope with Leica DFC290HD 3 MP color camera (Leica microsystems, Wetzlar, Germany) according to feasibility at different magnification.

### 4.6. Statistical Analysis

A total of 15 treatment combinations were evaluated for three different traits, and data were recorded for the upper and lower cotyledons of the immature zygotic embryos. Response surface models with a completely randomized design were selected, and regression models (linear, quadratic, and interaction terms) were fitted using a stepwise forward method. Significant factors (*p* < 0.05) were presented in the analysis of variance (ANOVA) summary, including the R-squared and adjusted R-squared values, the F-statistic, and *p*-value. Mean squares were calculated for each factor term. Data analysis and graphical representations were performed using JMP software version 16 (SAS Institute, Cary, NC, USA).

## 5. Conclusions

In this study, we developed a robust, highly efficient, and reproducible protocol for DSE in the peach rootstock cultivar Guardian^®^ using immature cotyledons and successfully regenerated whole plants via this approach. We optimized key parameters influencing somatic embryo induction, including the concentration of PGRs 2,4-D and KIN, as well as the developmental stage and size of zygotic embryos and the spatial orientation of cotyledons on preculture media. Among these, the concentrations of 2,4-D and KIN, along with cotyledon positioning, were identified as the most critical factors affecting embryogenic response. In addition to somatic embryos, we achieved consistent regeneration of secondary somatic embryos, which were induced more readily and could be maintained over extended periods through regular subculturing. The ability to propagate both somatic embryos and secondary somatic embryos establishes a continuous embryogenic culture system that is well-suited for downstream biotechnological applications. The somatic embryos generated through this system hold significant potential for advanced applications such as genome editing and RNA interference (RNAi) [34], in vitro screening for biotic and abiotic stress responses, and the production of haploid, double-haploid, and synthetic seeds [23]. The protocol presented here provides a foundational tool for enhancing peach rootstock improvement and will support the development of next-generation cultivars with desirable traits such as resistance to *Armillaria* root rot (ARR), continuous flowering, and enhanced cold tolerance. Moreover, this method may be extended and adapted to other peach genotypes, thereby advancing both basic research and applied breeding efforts in *Prunus* species.

## Figures and Tables

**Table 1:** SEIM combinations of different concentrations of 2,4-D and KIN.

Run	2,4-D (µM)	KIN (µM)
1	0.01	0.1
2	0.1	
3	0.3	
4	1	
5	3.2	
6	0.01	1
7	0.1	
8	0.3	
9	1	
10	3.2	
11	0.01	3.2
12	0.1	
13	0.3	
14	1	
15	3.2	

Note: The entire set of 15 treatments were tested on upper and lower cotyledons.

**Table 2:** Summary ANOVA of SE %, SE productivity and callus % in response to the 2,4-D and KIN.

(**A**) **Trait-SE % (RSquare = 0.81; RSquare Adjusted = 0.78)**			
Terms	Estimate	Mean Square	Prob > |t|
2,4-D × KIN	6.890537	6866.6806	<0.0001 ***
KIN	7.87553	3612.4906	<0.0001 ***
2,4-D	13.93409	3046.5154	<0.0001 ***
cotyledon location	−5.555556	1851.8519	0.0001 ***
KIN × cotyledon location	−3.193534	1037.5438	0.0031 **
2,4-D × 2,4-D	−4.108578	679.4956	0.0152 *
2,4-D × cotyledon location	−2.50891	536.9068	0.0300 *
(**B**) **Trait-SE productivity (RSquare = 0.52; RSquare Adjusted = 0.44)**			
Terms	Estimate	Mean Square	Prob > |t|
KIN	0.514562	15.421375	0.0001 ***
2,4-D	0.956029	14.341305	0.0002 ***
2,4-D × KIN	0.23283	7.840062	0.0050 **
2,4-D × 2,4-D	−0.408734	6.724904	0.0089 **
cotyledon location	−0.281667	4.760167	0.0264 *
(**C**) **Trait-Callus % (RSquare = 0.84; RSquare Adjusted = 0.82)**			
Terms	Estimate	Mean Square	Prob > |t|
2,4-D	40.17464	25,325.053	<0.0001 ***
cotyledon location	−16.66667	16,666.667	<0.0001 ***
2,4-D × 2,4-D	−13.92445	7804.766	<0.0001 ***
KIN	7.674595	3430.506	0.0004 ***
KIN × cotyledon location	−4.836173	2379.397	0.0025 **
2,4-D × KIN	−2.698459	1053.107	0.0390 *

***, **, and * designate the significance from a *t* test for each estimate at *p* < 0.001, 0.01, and 0.05, respectively.

## Data Availability

Data is contained within the article.

## Abbreviations

The following abbreviations are used in this manuscript:

DSE	Direct somatic embryogenesis
SE	Somatic embryogenesis
2,4-D	2,4-dichlorophenoxyacetic acid
KIN	Kinetin
µM	One millionth of a mole per liter
ISE	Indirect somatic embryogenesis
BAP	6-benzylaminopurine
PTSL	Peach tree short life
ARR	Armillaria root rot
QL	Quoirin and Lepoivre
MS	Murashige and Skoog
SEIM	Somatic embryogenesis induction media
PGRs	Plant Growth Regulators
DM	Differentiation media
NAA	1-Naphthaleneacetic acid
ANOVA	Analysis of variance
